# The Government Built It, and the Private Sector Came: For-Profit Health Care, Government Support, and the Road from Public Service to Private Equity

**DOI:** 10.1017/amj.2025.10070

**Published:** 2025-07

**Authors:** Arnold J. Rosoff, Robert I. Field, Anthony W. Orlando

**Affiliations:** Drexel University Thomas R. Kline School of Law, Philadelphia, PA, USA

**Keywords:** Health law, health insurance, Medicare, Medicaid, Private Equity, Health Technology

## Abstract

The catchphrase “if you build it, they will come,” from the movie “Field of Dreams,” described an audacious plan to build a small baseball stadium in a remote cornfield. It could also describe the government infrastructure which has drawn in the ever-growing American health care business sector. A series of increasingly complex and expensive programs, first launched just after World War II, continue to provide essential funding and regulatory support for a multitude of private companies that have revolutionized medical care and, in the process, built an industry that represents more than 18% of the country’s economy. This parade of programs includes the Hill-Burton Act of 1946, Medicare and Medicaid in 1965, the initiation of the Human Genome Project in 1990, and 2010’s Patient Protection and Affordable Care Act, all of which created platforms on which private entities rely to provide medical services and products. In the process, these private entities have made and continue to generate substantial profits. And while many of them have improved public wellbeing dramatically, many have also degraded the system’s integrity through fraud and anticompetitive behavior. In its role of keeping this huge and essential private enterprise on track, health law has become an indispensable part of the system, with health lawyers serving as the foundation of its effective operation.

## Introduction

“If you build it, they will come.” In the movie “Field of Dreams,” that phrase captured the expansive opportunity to attract people from far and wide simply by building an edifice in an unlikely, remote place. In the movie, the edifice in question was a primitive baseball stadium in an Iowa cornfield that would serve as an attraction for players and fans. But that phrase, and the promise behind it, also applies in other contexts — notably in American health care. In the health care context, the phrase encapsulates the trajectory of the industry over the past 75 years, with “you” being the federal government and “they” being the private health care industry. Over that time, the system has grown through an array of government funding and regulatory programs that created the field on which private entities, both domestic and foreign, have played the game of American health care business.

The government’s playing field and the private businesses it has attracted have nurtured health care as an ever-expanding, ever-more expensive public-private partnership. The result is a health care system that is more complex and costly than any other in the world.^1^ It is also a system that is extremely profitable for those who come to play. Health care was a $4.9 trillion industry in 2023,^2^ with the private sector estimated to account for about 30% of it.^3^ The health care industry which has developed over the past century now includes thousands of businesses serving millions of patients,^4^ with each business seeking the opportunity to share in the industry’s profits.

Many, although certainly not all, of the businesses are excellent players, enhancing the health of untold numbers of patients and giving the government substantial returns on its investments. However, even well-meaning players in the industry must navigate a bewildering maze of laws, regulations, and policies to enter and stay in the playing field. Health law has grown with the system as a critical component to guide private businesses and government policymakers.

Unfortunately, this complex playing field often, and with increasing frequency, gives those who are not good players the chance to undermine the system. This is seen, for example, in billing fraud under government health insurance programs, incentives for providers to favor wealthier and better-insured patients, commercialization of industry segments leading to market consolidation and higher prices, and corporatization of key provider sectors by private equity, which studies indicate can lead to poorer patient care.^5^ With regard to these businesses, the role of lawyers is especially critical in helping health care entities understand what the bounds of the law are and, often, where they can bend. Thus, the development and expansion of the field of health law is a key byproduct of the health care system’s evolution. On this field of dreams, games cannot be played without team owners to write the rules, coaches to teach them, and umpires to enforce them.

This article describes key developments in the evolution of American health care’s public-private partnership, the benefits and harms brought by these developments, and the increasingly central role of health lawyers. The public-private partnership has created an industrial juggernaut.^6^ In 2023, it represented more than one-sixth of the country’s economy.^7^ The range of enterprises it encompasses includes providers (both institutional and individual), insurers that finance provider services, and a host of ancillary health care businesses. As a result of the government support needed to sustain and oversee this growth, health care spending now devours the largest share of federal spending, eclipsing even the defense sector.^8^

## I. Origins and “Ancient” History

To understand any business, it is necessary to follow the money and see the forces that shape its incentives. In health care, most of that money comes from insurance, both public and private.^9^ Therefore, we begin our analysis with the origins of that financing mechanism.

### A. Private Health Insurance: Emergence and Regulation

Health insurance in the United States emerged in its present form in 1929, when Baylor University Hospital in Texas established the first Blue Cross plan to cover hospital costs.^10^ As similar plans were created in other parts of the country, Blue Cross’s network grew state-by-state throughout the Depression era and during World War II.^11^ The first Blue Shield plan was created in 1939 to cover the costs of physician care.^12^ It developed alongside the expanding array of Blue Cross plans to offer comprehensive coverage for medical expenses. The two plans were formally combined as the Blue Cross/Blue Shield Association in 1982.^13^ Both plans were committed to a nonprofit structure and to “community rating”, meaning that all individuals and companies enrolled in a plan were placed in the same risk pool and charged the same premium regardless of their individual or group loss experience.^14^ This practice distinguished these plans from those of private commercial plans, which actuarially rated individuals and groups they covered and charged them different premiums based on their risk characteristics.^15^

For almost half a century, the regulatory structure that oversaw health insurance was based on the Supreme Court’s 1869 decision in *Paul v. Virginia*,^16^ which ruled that insurance was not “interstate commerce” and, thus, not subject to federal regulation.^17^ As a consequence of that ruling, insurance regulation evolved at the state level, with each state administering its own regulatory apparatus.^18^ Over time, the state insurance regulators joined together to create the National Association of Insurance Commissioners (“NAIC”).^19^ When, in 1944, the Court overturned *Paul* in *U.S. v. Southeastern Underwriters Association*,^20^ the NAIC led a campaign to preserve state control over insurance, resulting in the 1945 passage of the McCarran-Ferguson Act.^21^ Under that law, authority to regulate health care was returned to the states. However, over subsequent decades, the federal government reasserted its authority in other aspects of the industry, eclipsing the states as the primary locus of oversight and policymaking in many respects.^22^ The result was a patchwork arrangement of state regulation, federal regulation, and business expansion.

### B. Private Health Insurance: The Foundation of Subsequent Government Financing

As discussed below, government programs to expand access to health care, such as Medicare and Medicaid, built upon the foundation of private financing rather than replacing it. The result is a hybrid structure like that overseeing private health insurance. This structure sees federal and state authorities cooperating to provide financing and oversight, while private entities provide much of the service and administration. Those private entities include Blue Cross/Blue Shield and a large number of commercial insurance companies.

To put this government insurance structure in historical context, it is helpful to understand the two prevailing national health care models that the United States could have followed. Under the Bismarckian approach — named after Otto Von Bismarck, Chancellor of the German Reich from 1871 to 1890 — the government requires private employers to provide health services for their employees and often for their family members as well.^23^ The underlying notion is that, in an effectively functioning society, employers and their employees form a core part of the economy, and therefore the government can use the employment structure to ensure that all — or, at least, most — citizens have a required minimum level of coverage. Under the Beveridge approach — promoted by Lord William Beveridge, who served in Parliament from 1944 to 1945 and helped form the basis of the British National Health Service — the national government both regulates and provides health services through its own employees and facilities.^24^

## II. America’s Path Toward More Universal Health Care: The Long and Winding Road With Bumps and Detours

### A. Truman Gets the Ball Rolling

The path toward broader, if not universal, coverage is strewn with a collection of programs based on the public-private partnership model. This path began in 1946, when Congress passed the Hill-Burton Act, an initiative of President Harry Truman that implemented the first major federal health care funding program.^25^ Unable to get a national health insurance plan through Congress, Truman accepted Hill-Burton as a “consolation prize” that would help private hospitals across the country build, expand, and modernize their facilities in order to serve more patients.^26^ In so doing, the federal government would correct for serious underinvestment in the health care system during the Depression and World War II.

Hill-Burton also addressed financial barriers to accessing care, although its funding for services has been inconsistent over the years.^27^ Moreover, in return for the chance to dip into the law’s six billion dollar pot of grants and loans, hospitals were required to comply with a set of regulations related to access, including nondiscrimination rules, which represented the first direct federal oversight of the industry.^28^ With that platform of funding and regulation, the modern hospital system was born.

### B. Medicare and Medicaid and a Federal “Blank Check” to the Health Care Industry

Truman’s vision came closer to realization two decades later with the creation of Medicare and Medicaid in 1965. Although preceded by some less-ambitious initiatives,^29^ these were the first major federal programs to broadly address financial barriers to care.^30^ While Truman’s goal of universal coverage was not achieved, coverage was guaranteed for some groups, namely the elderly, disabled, and some categories of the poor.^31^ Medicaid, passed into law in 1965 along with Medicare, relies on a federal-state partnership model which uses a “carrot and stick” approach, combining incentives for participating and disincentives for abstaining. Under Medicaid, the federal government offers money to help states finance health care for the poor if they meet and adhere to federal program standards.^32^ Medicare, by contrast, is a unified federal program, originally administered by the Social Security Administration.^33^ Even with this structure, though, Medicare has a strong private sector component, in which the administration of claims is carried out to a large extent through private companies under contract with the federal government.^34^

A revealing footnote to the birth of Medicare is that the legislative proposal was hurriedly penned in July of 1965 by Wilbur Cohen, Secretary of the Department of Health, Education and Welfare, and one of his chief aides, William Kissick.^35^ The Cohen-Kissick draft, which formed the core of the Social Security Amendments of 1965 that established the program, was written in response to a Thursday afternoon call from President Lyndon Johnson, who had arranged for a Congressional sponsor to introduce the bill the following Monday and needed a proposal as soon as possible.^36^ Blue Cross and Blue Shield were used as the template because their networks accounted for more than fifty percent of the health insurance market in the United States.^37^ The premise behind doing so was that the federal government would simply pick up the tab for that predominant form of coverage for the nation’s elderly and disabled — a private template for federal infrastructure.

This approach was born out of political necessity, as the American Medical Association, along with other prominent health care organizations, had long waged a vigorous fight against what they termed “socialized medicine,” meaning government influence over the health care system.^38^ This accounts for the stark language of the opening section of the Medicare law, which forbids federal employees and officers from controlling or interfering with the practice of medicine.^39^ The law also avoided all but minimal consideration of cost control.^40^ That situation changed markedly over subsequent decades.

An important and sometimes overlooked aspect of Medicare and Medicaid is the extent to which they were designed not to operate independently of the private sector. Despite the frequent mischaracterization of Medicare as a national “single payer” program, the government has built a structure in which private companies can and do play a prominent role.^41^ Private hospitals and physicians, not government providers, continue to render care, and financing remains a shared responsibility. Private insurance companies continue to sell policies supplementing Medicare, in addition to administering claims through subsidiaries in each state.

The federal government’s role in overseeing this complex arrangement fell to an expanding bureaucracy. Although Section 1801, forbidding federal control of medical practice,^42^ remains in the statute, it became increasingly clear that some degree of oversight and cost control was needed. As discussed below, Congress passed a package of Medicare amendments in 1972, enabling the Social Security Administration to take the lead in administering new regulations. By 1977, the regulatory demands had grown so complex that a separate agency was needed, and so the Health Care Financing Administration (“HCFA”) was created. With the addition of Medicare Advantage under Part C of the program, along with prescription drug coverage under Part D in 2003 (enacted as part of the Medicare Modernization Act), the agency’s role grew again, and it was given a new name, the Centers for Medicare & Medicaid Services (“CMS”).^43^

Health care providers, insurance companies, and consumers had to keep up with this growing maze of bureaucracy. In response, health law as a distinct field grew in prominence, and, beginning in the 1970s, legal professionals and scholars formed a number of academic and professional organizations focusing on health law and policy. Notable organizations included the American Health Law Association (“AHLA”), the American College of Legal Medicine, and the American Society of Law and Medicine (“ASLM”), now known as the American Society of Law, Medicine and Ethics (“ASLME”).^44^

### C. The First Major Medicare Amendments in 1972

As Cohen and Kissick had anticipated, the inauguration of Medicare and Medicaid greatly increased the demand for health care services and the country’s total expenditures on such services.^45^ Leaving it to physicians to determine the need for health services on a case-by-case basis, consistent with the Blue Cross model, made the early Medicare structure the very antithesis of cost containment. With the government standing by as a generous payor, hospitals and the doctors practicing in them had a substantial incentive to keep patients in the hospital longer to generate more revenue for the institution. As UCLA Professor Milton Roemer famously observed with regard to hospitals in 1968, “a bed built is a bed filled” under fee-for-service payment.^46^ Recognition of this cost-increasing effect came to be known as “Roemer’s Law.”^47^

Clearly something had to be done. A first step was to control the overbuilding of hospital beds. Doing this required consensus standards for how long a patient should stay in the hospital for a particular condition. This was addressed through Certificate of Need (“CON”) laws in a number of states and, ultimately, the passage of the federal National Health Planning and Resources Development Act, which divided the country into Health Service Areas (“HSAs”).^48^ Under CON laws, hospitals wishing to expand their facilities had to show statistically that this expansion was actually needed — in other words, new government oversight in return for the continued flow of government dollars.

However, another step was needed for the physician side to control excessive lengths of stay during which an admitting physician could order and bill for the services they rendered in the hospital. This was addressed through the 1972 amendments by creating a nationwide network of Professional Standards Review Organizations (“PSROs”), regional organizations that mobilized the medical community to articulate and enforce standards regarding the medical necessity and reasonableness of services delivered and paid for through Medicare.^49^ Those standards were then used to monitor physician patterns of utilizing services and resources. Even with the intervention of PSROs, however, there remained a substantial variety of ways to treat a patient identified as suffering from a given medical condition, some more expensive than others. PSROs were a far cry from an enforceable uniform national standard.

As Roemer’s law applies mainly to fee-for-service reimbursement, in which each service rendered generates revenue for the provider, policymakers began to realize that this kind of reimbursement was at the heart of run-away costs. This led to an effort to switch payment under Medicare and Medicaid to a “prospective payment” model, under which reimbursement is paid based on a patient’s condition rather than the number of services rendered to treat it. This led to a move to pay hospitals for inpatient stays on the basis of Diagnosis-Related Groups (“DRGs”), sets of diagnoses that required similar sets of services.^50^ Under this approach, statistics are developed on the set of services that hospitals provide, including the number of days of hospitalization customarily used to treat a particular diagnosis.^51^ If attending physicians feel that a patient’s situation justifies deviating from the standard package, it is incumbent on them to marshal the evidence to prove the need for extra payment. The DRG approach was first tried as a state-wide experiment under New Jersey’s hospital rate-setting system in 1980 and then extended to Medicare in 1983.^52^ Nevertheless, while the government changed the rules of reimbursement, hospitals continued to find new ways to play and win at the game of revenue generation.^53^

## III. Broader Federal Infrastructure and the Birth of Managed Care

### A. The Managed Care Movement

The prospective payment concept revolutionized not just Medicare and Medicaid, but also American health care more generally. Its most basic form is the Health Maintenance Organization (“HMO”), a health care delivery system developed by Dr. Paul Ellwood at Interstudy, a Minneapolis-based health policy think tank he founded and directed.^54^ In HMO plans, patients are assigned a designated primary care physician who directs all their care, subject to oversight by the plan.^55^ In 1973, the federal HMO Act was passed, offering subsidies to fund the startup and initial operation of nonprofit HMOs and mandating that larger employers who provide health insurance to their employees offer an HMO or “dual choice” alternative.^56^ As the HMO movement took off, HMOs evolved in two main forms. Prepaid Group Practices (“PGPs”), follow the model of Kaiser-Permanente in which the plan directly employs physicians and owns its own hospitals.^57^ The other form, Individual Practice Associations (“IPAs”), use networks of private independent practices.^58^ Over time, more variants emerged, including Preferred Provider Organizations (“PPOs”), and a more inclusive term, Managed Care Organizations (“MCOs”), was applied to cover all of them.^59^ In the early 1980s, an increasing number of HMOs switched from nonprofit to for-profit status, and the for-profit structure soon came to dominate the market.^60^

Subsequent decades saw HMOs evolve into yet another private health care sector founded and shaped by federal policies. They grew from a humble beginning where they needed a federal push to get off the ground into the predominant form of private health insurance in the United States. From 1976, three years after the Act was passed, until 1987, the number of plans increased from 175 to 650, and the number of enrolled members increased from six million to 29.3 million.^61^

HMOs also received an unexpected boost from the Employee Retirement Income Security Act of 1974 (“ERISA”), a Byzantine law that eased the path to self-insurance for large employers.^62^ Under that law, many of those companies could choose to cover their employees’ health care costs themselves without going through an insurance company, and many chose to do so by contracting with MCOs.^63^ This meant that companies could save on administrative costs by cutting out insurance companies as middlemen and offer managed care plans to their workers as a cost-saving, at least in theory, approach to coverage. Along with self-insurance under ERISA came a new health care regulatory player, the Department of Labor (“DOL”),^64^ expanding the overall health care regulatory playing field.

Dominating the private insurance market was just the start. In 1997, MCOs received another boost from federal policy when Medicare added Part C (now known as Medicare Advantage) as an option for beneficiaries to replace traditional coverage with a private managed care plan.^65^ In 2006, Medicare Part D, which uses private plans to provide prescription drug coverage, took effect.^66^ State policy also provided a major boost. Starting in the 1980s, several states began to administer Medicaid through private managed care plans, and today all states do so to at least some extent.^67^ In 2016, Medicare and Medicaid accounted for $213.1 million, or 59%, of revenue from premiums and administrative-services-only contracts across the five largest health insurance companies.^68^

### B. Expanding Access Through the Affordable Care Act

In 2010, the most complex federal health care infrastructure program of all was created by the Patient Protection and Affordable Care Act, more commonly known as the Affordable Care Act (“ACA”) and often referred to as “Obamacare.”^69^ It expanded coverage in two main ways. First, it created online exchanges through which Americans could purchase individual coverage without regard to preexisting medical conditions and with subsidies for those with lower incomes.^70^ Second, it offered states a financial incentive to expand their Medicaid programs to cover more people.^71^

Both of these measures gave the private sector huge new opportunities. The exchanges create a market for private individual insurance with millions of new customers, while the expansion of Medicaid means a larger role for MCOs that administer plans on behalf of states.^72^ The greater number of insured individuals, in turn, generated more patient demand for the services of hospitals and other providers to the point where many institutions depend on Medicaid and other government subsidies for their very financial survival.^73^ These measures also vastly expanded the role and responsibilities of CMS, not to mention the demand for lawyers to work in it, with it, and against it.^74^ Despite numerous attempts to repeal it, the ACA is now more than fourteen years old, stable, and continuing to move the United States closer to its ideal of universal health coverage.^75^

## IV. Private Health Care Grows from Government Sidekick to Big Business

### A. For-Profit Proliferation

Beyond insurance companies, a range of private players — including hospitals, physicians, and nursing homes — have built a web of profitable initiatives on the government’s playing field. Investors regularly demonstrate their awareness of this interdependence between private players and government support. Notably, the stock prices of publicly traded health care companies, which reflect expectations of future profits, often rise and fall alongside Medicaid enrollment.^76^ In fact, during the COVID-19 pandemic, one of the best predictors of a hospital’s financial resilience was its home state’s decision to join the ACA Medicaid expansion.^77^ Moreover, for-profit entities throughout the industry have come to rely on a wide range of government subsidies — from temporary support under the CARES Act passed in the early stages of the COVID-19 pandemic to various tax breaks to Medicare and Medicaid reimbursement — without a requirement to provide charity care or other public services commensurate with the public support they receive.^78^

More than simply helping a subset of players in the health care industry, government support has fueled a broader corporatization of health care by freeing capital for mergers and acquisitions that have consolidated providers and reduced competition in many local markets across the country. In 2005, only 53% of hospitals belonged to a larger system.^79^ By 2017, those systems accounted for 66% of all hospitals.^80^ This consolidation often empowers systems to act monopolistically, restricting access to health care and driving up the prices that consumers pay.^81^ There has also been substantial vertical integration among health care systems, allowing hospitals to refer their patients to more expensive physicians within the same system.^82^ When a system’s market share becomes large enough, it can also gain monopsonistic power over labor markets in which physicians seek employment.^83^

In concentrated markets, physicians’ satisfaction with their jobs tends to decline, as health system employers and insurance companies often force them to spend less time with each patient, generating more stress for the physician and worse outcomes for the patient.^84^ The same trend has been found in hospice care, where for-profit institutions cut costs to boost their bottom line and therefore score significantly lower on patient satisfaction measures.^85^ For-profit entities are also more likely to shift their treatment mix to emphasize more profitable procedures. When these hospitals experience financial losses, the incentive to do so is even greater, often to the neglect of other treatments their patients may need.^86^

### B. The Growing Role of Private Equity in Health Care

Corporate health care has proven so profitable that it has drawn new investors from outside the industry. Large institutional investors, such as pension funds and university endowments, are always looking for new industries in which to invest, but they do not typically possess the internal expertise or management capacity to play a controlling role in health care firms.^87^ This has created an opportunity for private equity funds — insular, non-public funds which pool investments from wealthy, sophisticated investors in order to acquire entities — to seek to fundamentally restructure these firms with the goals of realizing short-term profits and outsized returns for their investors.^88^ They have channeled large amounts of institutional capital into the health care industry, with fund managers taking on the task of restructuring the target firms.^89^

Contrary to the private equity sector’s claims, these firms are rarely in the business of saving health care entities from financial distress. Research has shown that private equity investors more often target hospitals with better financial performance that are not in need of rescue.^90^ Rather than trying to improve performance, the new owners often break acquired hospitals into pieces, strip assets from them, and sell the pieces to separate owners, under the theory that the market will pay more for them if they are put to a different use.^91^ An especially profitable piece is often the facility’s real estate, which may have value for a range of different businesses. The net effect is often to take much of the hospital industry, and other institutional providers such as nursing homes, out of the business of serving their patients.

This process often hollows out the facilities so significantly that they have no choice but to file for bankruptcy.^92^ This outcome may seem like a failure, but private equity firms often do not count on the success of a provider to generate their returns. Instead, they have often siphoned off the profitable assets, allowing them to walk away without incurring much cost and with a strong short-term return on their investment.^93^ The ironic upshot of this scheme is that the government’s health care infrastructure has been used to diminish, rather than enhance, the country’s health care capacity.

For the providers that survive private equity takeovers, the delivery of health care can change dramatically. This results from three strategies private equity owners use to generate high returns. First, they cut costs, typically by reducing staffing levels and dispensing with costly safety protocols. Patients have been found to experience a twenty-five percent increase in adverse events as a result.^94^ Second, they minimize the treatment of low-income patients, focusing instead on more profitable customers.^95^ Third, they “roll up” physician practices, creating market power by bringing many smaller entities that had been competitors under one umbrella.^96^ This consolidation allows them to charge higher prices, which then leads insurance companies to charge higher premiums.^97^ Research has found that higher insurance costs can create severe financial strain for small companies, and, in some instances, cause them to discontinue contributing to employee health insurance premiums altogether.^98^

Yet, with all of this corporate activity, “private” equity may be a misnomer. The companies may not be publicly traded or publicly owned, but they rely heavily on public subsidies. Many of their largest investors, including public pension funds and university endowments, enjoy large tax breaks because they theoretically serve a public purpose.^99^ Their acquired providers, including hospitals and physician practices, receive reimbursement from private health insurance that is publicly subsidized through its tax treatment or from government programs, such as Medicare and Medicaid. Even the private equity fund managers themselves pay lower taxes than employees of other firms, as investment activities are taxed at a lower rate than wages.^100^ At the same time, many patients still pay higher prices that the acquired entities charge.^101^

## V. The Playing Field for Health Care Products

### A. Drugs and Devices

While the provider and finance sides of health care were coming in waves to play in the government’s field of dreams, the pharmaceutical industry was playing just as aggressively. After World War II, the National Institutes of Health (“NIH”) became indispensable to financial support for the basic biomedical research that forms the backbone of new drug development.^102^ On top of that, the Human Genome Project (“HGP”), launched in 1990, led to a new era in therapeutics in the form of genomic medicine and precision medicine, through which drugs and treatment modalities are tailored to an individual patient’s genetic makeup.^103^ As American medicine’s pharmacopeia has grown, intellectual property protection for the industry’s products has become an ever more pressing concern — and with it, the role of the federal Patent and Trademark Office, which decides which products deserve protection from competition.^104^ Further, the widening scope of pharmaceutical interventions has imposed larger and more complex responsibilities on the Food and Drug Administration (“FDA”) in overseeing the testing, use, and marketing of new drugs and devices.^105^

### B. Health Technology

#### 1. Mobile Health

Alongside the government’s investments in pharmaceuticals over the past seventy-five years, its investments in technology have generated a revolution in our society, a revolution now reaching into every facet of health care. The computer, the cell phone, and all of Silicon Valley’s other products and services would not exist in their current advanced state without large, early inflows of tax dollars.^106^ Most of our digital and mobile technologies began in government-funded basic research laboratories and grew into profitable enterprises thanks to the government’s role as one of the first and largest purchasers of these technologies.^107^ It was only a matter of time before these for-profit enterprises figured out how to use the same digital and mobile technologies to deliver and finance heath care.

Today, health care consumers can open their phone and find thousands of health-related apps to download.^108^ They can order at-home tests for everything from COVID-19 to cholesterol to food sensitivity to fertility to sleep apnea.^109^ They can track their heart rate, respiratory rate, skin temperature, blood pressure, and even their glucose levels with watches, cell phones, and other wearable devices.^110^ They can even scan their own brain activity with at-home electroencephalogram headsets.^111^ Some of these devices are regulated by the FDA. Many are not. Few are used with the supervision of a medical professional.^112^

Even medical professionals are taking advantage of the convenience and time savings afforded by digital health care delivery. “Telehealth” (or “telemedicine”) has been growing for decades, first with phone call appointments and now with video calls. The use of telehealth further accelerated during the COVID-19 pandemic because it allowed patients and physicians to connect at a distance.^113^ The measured impact on patient health has been mostly positive. Access to telehealth is associated with fewer emergency room visits and better adherence to medication regimens.^114^ Patients are only slightly more likely to return for an in-person follow-up visit after a telemedicine appointment, compared to an initial in-person visit.^115^ However, many outcomes remain unmeasured, many populations remain underserved, and the telehealth profit motive often crowds out other services that cannot be delivered digitally. It remains to be seen whether this technology will disrupt more than it improves health care.^116^ In either event, this telehealth revolution represents another innovation generated by collaboration between government infrastructure and private sector initiative.

Health law has struggled to keep up with these fast-and-furious developments. The FDA has shied away from defining most at-home technologies as “medical devices,” and Congress has signaled its support for this hands-off approach.^117^ Therefore, as a growing share of health care is delivered outside of the traditional medical facility setting, patients will find that their health care is increasingly unregulated — and their privacy, safety, health, and civil rights are increasingly unprotected.^118^ This creates a gap yearning for a new kind of health law to fill.

#### 2. Artificial Intelligence

Nowhere is the technological frontier expanding more rapidly than in the use of artificial intelligence (“AI”). In clinical settings, evidence has already shown that AI can often diagnose diseases more accurately and prescribe treatments more effectively than human physicians.^119^ In the laboratory setting, researchers are finding that AI can process large datasets, sequence genomes, find useful connections and correlations, and identify “precision medicine” treatments targeted to specific individuals far more quickly and accurately than previous statistical models.^120^ AI care managers are even being employed in place of nurses and physician assistants to deal with patients by phone or video conferencing, for example, in routine follow-up care.^121^ We are only beginning to understand how these advances will transform almost every aspect of the industry.

At the same time, AI presents a range of new challenges for lawyers, regulators, and policymakers. Because AI models require large datasets to achieve many of their objectives, their widespread development is constrained by the willingness and ability of researchers to share data across institutions. This data-sharing, in turn, raises significant privacy risks. Yet many institutions do not have the necessary protections in place to guard against these risks.^122^ Meanwhile, even when the data are shared, stored, and used properly, investigations have revealed that AI models often deliver inequitable results that favor certain subgroups of the population over others.^123^

The future of AI in health care is a quintessential example of a health care evolution in need of guidance from federal policy. While Congress has yet to pass legislation to protect Americans from possible threats, the federal government has funneled billions of dollars into the AI industry through funding of research and development.^124^ Further, through its role as one of the earliest and largest customers for AI products, the government is fueling the growth of the industry even further as it spreads into every aspect of health care.^125^ Never let it be said that the private sector built our new AI-infused health care system alone. It is now, and will continue to be, a transformation that was built, as all health care transformations of the past seventy-five years have been, on the playing field the government built and continues to oversee.

## Conclusion

All of the key developments in the evolution of health care, from health insurance to hospital growth to pharmaceuticals to AI, have seen the private sector use government infrastructure to achieve remarkable advances. At the same time, new variants of health care business have led to many of the same problems that have plagued the industry since its dawn. Fraud, abuse, profiteering, and inequitable access to diagnosis and treatment have reared their heads at every step. These threats to the health care system’s integrity are now joined by intrusions into patient privacy and unaccountable technologies, such as AI. Health lawyers have time and again stepped into the breach to help oversee and control these behaviors in a never-ending battle for safety, efficacy, fairness, justice, and the protection of patients’ rights. The profit motive does not care about such matters. It barrels forward toward whatever consumers — and government programs — will pay for, often indifferent to non-pecuniary consequences.^126^ The government’s field of dreams created modern health care, but it has been the job of health lawyers to work to keep it level and to secure its benefits for everyone — a job that will become increasingly more essential and challenging as the game evolves.

## Footnotes

1
*See How Does the U.S. Healthcare System Compare to Other Countries?*, Peter G. Peterson Found. (Aug. 16, 2024), https://www.pgpf.org/article/how-does-the-us-healthcare-system-compare-to-other-countries/ [https://perma.cc/V5QE-USEP]; *see also* Jafar Sadegh Tabrizi et al., *Public-Private Partnership Policy in Primary Health Care: A Scoping Review*, 11 J. Primary Care & Cmty. Health (2020), https://journals.sagepub.com/doi/10.1177/2150132720943769.

2
*How Does Government Healthcare Spending Differ from Private Insurance?*, Peter G. Peterson Found. (Apr. 11, 2025), https://www.pgpf.org/article/how-does-government-healthcare-spending-differ-from-private-insurance/ [https://perma.cc/GR5E-C6M6].

3
*Id.*

4
*See County Business Patterns 2022*, U.S. Census Bureau, https://data.census.gov/table/CBP2022.CB2200CBP?q=CBP2022.CB2200CBP&codeset=naics~N0300.62 (last visited May 25, 2025) (reporting 678,288 ambulatory health care services establishments, 7,713 hospitals, and 95,137 nursing and residential care facilities establishments).

5
*See, e.g.*, Erin C. Fuse Brown & Mark A. Hall, *Private Equity and the Corporatization of Health Care*, 76 Stan. L. Rev. 527, 544–45 (2024); Zachary Levinson et al., *Ten Things to Know About Consolidation in Health Care Provider Markets*, KFF (Apr. 19, 2024), https://www.kff.org/health-costs/issue-brief/ten-things-to-know-about-consolidation-in-health-care-provider-markets/ [https://perma.cc/K84C-JVZX]; Meredith Freed et al., *Medicare Program Integrity and Efforts to Root Out Improper Payments, Fraud, Waste and Abuse*, KFF (Mar. 31, 2025), https://www.kff.org/medicare/issue-brief/medicare-program-integrity-and-efforts-to-root-out-improper-payments-fraud-waste-and-abuse/ [https://perma.cc/JB23-G2SQ].

6 Robert I. Field, Mother of Invention: How the Government Created “Free-Market” Health Care 41–42 (2014).

7 Anne D. Martin et al., *National Health Expenditures in 2023: Faster Growth as Insurance Coverage and Utilization Increased*, 44 Health Affs. 12, 13 (2025).

8 Juliette Cubanski et al., *What Does the Federal Government Spend on Health Care?*, KFF (Feb. 24, 2025), https://www.kff.org/medicaid/issue-brief/what-does-the-federal-government-spend-on-health-care/ [https://perma.cc/7BJR-BLCE].

9
*See* Peter G. Peterson Found., *supra* note 2.

10 Paul Starr, The Social Transformation of American Medicine 295–98 (2d ed. 2017).

11
*Id.* at 295–98, 308.

12
*Id.* at 306.

13 Blue Cross & Blue Shield Ass’n, BCBSA History Fact Sheet 2 (1997), https://digitalcommons.unf.edu/cgi/viewcontent.cgi?article=3089&context=flablue_text [https://perma.cc/82A8-SWM2].

14
*See* Starr, *supra* note 10, at 328–30.

15
*See id*; Arnold J. Rosoff & Anthony W. Orlando, *Employers and Health Insurance Under the Affordable Care Act*, 24 Annals Health L. 470, 472 & n.10 (2015).

16
*See* Paul v. Virginia, 75 U.S. (7 Wall.) 168, 183 (1869).

17
*See id.*; U.S. Const. art. I, § 8, cl. 3.

18 Baird Webel, Cong. Rsch. Serv., R43067, Insurance Regulation: Issues, Background, and Legislation in the 113th Congress 21 (2015).

19
*See id.* at 16; *See 150th Anniversary*, Nat’l Ass’n of Ins. Comm’rs, https://content.naic.org/150-timeline [https://perma.cc/M4Y5-ZYCB] (last visited May 10, 2025).

20 United States v. South-Eastern Underwriters Ass’n, 322 U.S. 533, 553 (1944).

21
*See* Webel, *supra* note 18, at 16; *McCarran-Ferguson Act: Background*, Nat’l Ass’n of Ins. Comm’rs (July 3, 2024), https://content.naic.org/insurance-topics/mccarran-ferguson-act [https://perma.cc/8KRG-N7YA] (“To remove any uncertainty over states’ regulatory authority over insurance created by that decision, the NAIC proposed a bill sponsored by U.S. Senators Pat McCarran (D-NV) and Homer S. Ferguson (R-MI) that would keep insurance regulation in the hands of the states.”).

22
*See* Webel, *supra* note 18, at 16–23.

23 Theodore H. Tulchinsky, Case Studies in Public Health 141–42 (2018).

24
*Id.* at 142–44.

25
*Hill-Burton Free and Reduced-Cost Health Care*, Health Res. & Servs. Admin. (Sept. 2023), https://www.hrsa.gov/get-health-care/affordable/hill-burton [https://perma.cc/DP7V-N87H].

26
*See* Starr, *supra* note 10, at 280–89.

27
*See, e.g.*, *Hill-Burton Free and Reduced-Cost Health Care*, *supra* note 25.

28
*See generally* U.S. Comm’n on C.R., Equal Opportunity in Hospitals and Health Facilities: Civil Rights Policies Under the Hill-Burton Program (1965), https://www.nlm.nih.gov/exhibition/forallthepeople/img/1706.pdf. The nondiscrimination rules permitted “separate but equal” facility resources until such practice was found unlawful in 1963. Simkins v. Moses H. Cone Mem’l Hosp., 323 F.2d 959, 970–71 (4th Cir. 1963).

29
*See* Judith D. Moore & David G. Smith, *Legislating Medicaid: Considering Medicaid and Its Origins*, Health Care Fin. Rev., Winter 2005, at 45, 45–47 (describing the Kerr-Mills Act and other early welfare-based medical payment programs).

30
*See* Starr *, supra* note 10, at 369; Darren A. DeWalt et al., *Significance of Medicare and Medicaid Programs for the Practice of Medicine*, Health Care Fin. Rev., Winter 2005, at 79.

31
*See* Alison Mitchell et al., Cong. Rsch. Serv., R43357, Medicaid: An Overview 5 (2025); Paulette C. Morgan & Phoenix Voorhies, Cong. Rsch. Serv., IF10885, Medicare Overview 1 (2024).

32 Elizabeth Williams et al., *Medicaid Financing: The Basics*, KFF (Jan. 29, 2025), https://www.kff.org/medicaid/issue-brief/medicaid-financing-the-basics/ [https://perma.cc/QB3V-42HL].

33
*See An Overview of Medicare*, KFF (Feb. 13, 2019), https://www.kff.org/medicare/issue-brief/an-overview-of-medicare/ [https://perma.cc/UD5S-7NMK]; Morgan & Voorhies *, supra* note 31, at 1–2.

34
*See* Stanley B. Jones, *Medicare Influence on Private Insurance: Good or Ill?*, Health Care Fin. Rev., Winter 1996, at 153, 153–54; Meredith Freed et al., *Medicare Advantage in 2024: Enrollment Update and Key Trends*, KFF (Aug. 8, 2024), https://www.kff.org/medicare/issue-brief/medicare-advantage-in-2024-enrollment-update-and-key-trends/ [https://perma.cc/BM9Q-BQ2W].

35 Willam Kissick was one of two physicians who collaborated with Secretary Cohen in drafting the Medicare legislation. One of this article’s authors (Rosoff) recalls Dr. Kissick’s telling countless times the story of how he sat at Cohen’s dinette table over a weekend to get a draft in President Johnson’s hands “ASAP.” *See also* Wilbur J. Cohen, *Reflections on the Enactment of Medicare and Medicaid*, 1985 Health Care Fin. Rev. (Ann. Supp.) 3, 6.

36
*See supra* note 35; *see also Lyndon B. Johnson Tapes: Conversation WH6507.04*, Soc. Sec. Admin. (Sept. 2, 1964), https://www.ssa.gov/history/LBJ/lbj.html [https://perma.cc/H6AU-ZYGS].

37
*See* Nancy De Lew, *Medicare: 35 Years of Service*, Health Care Fin. Rev., Fall 2000, at 75, 77.

38
*See* Starr, *supra* note 10, at 280–83, 299–307.

39 Social Security Amendments of 1965, Pub. L. No. 89-97, § 1801, 79 Stat. 286, 291 (codified at 42 U.S.C. § 1395).

40 As Kissick related it, he asked Secretary Cohen if there shouldn’t be utilization and cost controls written into the proposal and Cohen replied to the effect that “Shh, don’t even mention cost containment; that will kill the bill. Let’s get it passed and we’ll cross that bridge when we come to it.” *See supra* note 35. LBJ signed the bill into law on July 30, 1965, and we’ve been “crossing that bridge” ever since, struggling to control the dramatic healthcare cost spiral. Historical health expenditure data are available at CMS.gov. *See National Health Expenditure Data: Historical*, Ctrs. for Medicare & Medicaid Servs. (Dec. 18, 2024), https://www.cms.gov/data-research/statistics-trends-and-reports/national-health-expenditure-data/historical [https://perma.cc/Q3MW-9ZJH].

41 Linda J. Blumberg & John Holahan, Urb. Inst., The Pros and Cons of Single-Payer Health Plans 2 (2019), https://www.urban.org/sites/default/files/publication/99918/pros_and_cons_of_a_single-payer_plan.pdf [https://perma.cc/9EEQ-88H5].

42 Social Security Amendments of 1965, Pub. L. No. 89-97, § 1801, 79 Stat. 286, 291 (codified at 42 U.S.C. § 1395).

43 Thomas G. McGuire et al., *An Economic History of Medicare Part C*, 89 Milbank Q. 289, 290, 308–09, 313–14 (2011).

44
*See Who We Are*, Am. Soc’y L., Med. & Ethics, https://aslme.org/about-us/ [https://perma.cc/R3PF-SHB7] (last visited Dec 19, 2024); *About*, Am. Coll. of Legal Med., https://aclm.org/about/ [https://perma.cc/L379-VY34] (last visited May 10, 2025); *About AHLA*, Am. Health L. Ass’n, https://www.americanhealthlaw.org/about-ahla [https://perma.cc/Y3JU-MQPC] (last visited May 10, 2025).

45 Matthew McGough et al., *How Has U.S. Spending on Healthcare Changed over Time?*, Health Sys. Tracker (Dec. 20, 2024), https://www.healthsystemtracker.org/chart-collection/u-s-spending-healthcare-changed-time [https://perma.cc/QU8C-UK5K].

46 Paul L. Delamater et al., *Do More Hospital Beds Lead to Higher Hospitalization Rates? A Spatial Examination of Roemer’s Law*, 8 PLOS One art. no. e54900, at 1, 14 (2013).

47
*See, e.g.*, *id.*

48
*See* Matthew D. Mitchell, *Certificate of Need Laws in Health Care: Past, Present, and Future*, 61 Inquiry art. no. 469580241251937, at 1–2 (2024) (discussing the history of CON laws); National Health Planning and Resources Development Act of 1974, Pub. L. No. 93-641, § 1511, 88 Stat. 2225, 2229 (codified as amended at scattered sections of 42 U.S.C.) (establishing HSAs federally).

49 Social Security Amendments of 1972, Pub. L. No. 92-603, 86 Stat. 1329.

50
*See* Starr, *supra* note 10, at 454.

51
*See generally* Ctrs. for Medicare & Medicaid Servs., PBL-038, Design and Development of the Diagnosis Related Group (DRG) (2019).

52 William C. Hsiao et al., *Lessons of the New Jersey DRG Payment System*, 5 Health Affs. 32, 33 (1986).

53
*See* Hyunmin Kim et al., *Hospital Value-Based Payment Programs and Disparity in the United States: A Review of Current Evidence and Future Perspectives*, 10 Frontiers Pub. Health art no. 882715, at 1, 17–19 (2022).

54 Michael A. Morrisey et al., *Hospitals and Health Maintenance Organizations: An Analysis of the Minneapolis-St. Paul Experience*, Health Care Fin. Rev., Spring 1983, at 59, 63.

55
*Health Maintenance Organization (HMO)*, HealthCare.gov, https://www.healthcare.gov/glossary/health-maintenance-organization-hmo/ [https://perma.cc/JZ5A-QRHV] (last visited Feb. 26, 2025).

56 Health Maintenance Organization Act of 1973, Pub. L. No. 93-222, 87 Stat. 914; *id.* §§ 1303–04, 87 Stat. at 920–25 (codified as amended at 42 U.S.C. §§ 300e-2 to -3, repealed by Drug Export Amendments Act of 1986, Pub. L. No. 99-660, § 803(a), 100 Stat. 3743, 3799) (authorizing the Secretary to make grants to and enter contracts with public or nonprofit private entities for purposes of surveying feasibility of, planning establishment of, initially developing, or expanding HMOs); *id.* § 1305(a), 87 Stat. at 924 (codified as amended at 42 U.S.C. 300e-4(a)) (authorizing the Secretary to make loans to public or nonprofit private HMOs); *id.* § 1310(a), 87 Stat. at 930 (codified as amended at 42 U.S.C. § 300e-9(a)) (requiring employers with twenty-five or more employees to include in their health benefits plans the option membership in a qualified HMO); *see also* Joseph L. Dorsey, *The Health Maintenance Organization Act of 1973 (P.L. 93-222) and Prepaid Group Practice Plans*, 13 Med. Care 1, 1–9 (1975).

57
*See, e.g.*, Arnold J. Rosoff, *Phase Two of the Federal HMO Development Program: New Directions After a Shaky Start*, 1 Am. J.L. & Med. 209, 216 (1975).

58
*See id.*

59
*See* Kimberley H. Geissler et al., *The Association of Insurance Plan Characteristics with Physician Patient-Sharing Network Structure*, 21 Int’l J. Health Econ. & Mgmt. 189, 189–201 (2021); Amber Ratcliffe, *What Is a Managed Care Organization (MCO)?*, MedTrainer, https://medtrainer.com/blog/managed-care-organization/ [https://perma.cc/6MGL-BE37] (last visited May 10, 2025).

60 Kathryn M. Langwell, *Structure and Performance of Health Maintenance Organizations: A Review*, Health Care Fin. Rev., Fall 1990, at 71, 73; *see also* Omnibus Budget Reconciliation Act of 1981, Pub. L. No. 97-35, § 943, 95 Stat. 357, 576 (striking out the “nonprofit” limitation in the Secretary’s authority to issue loans to HMOs); Ellen M. Morrison & Harold S. Luft, *Health Maintenance Organization Environments in the 1980s and Beyond*, Health Care Fin. Rev., Fall 1990, at 81, 84 (“In 1983, Wall Street began to favor the HMO industry, and many not-for-profit HMOs changed their status to for-profit.”).

61 Lynn R. Gruber et al., *From Movement to Industry: The Growth of HMOs*, 7 Health Affs. 197, 198–99 (1988).

62
*See* Employee Retirement Income Security Act of 1974, Pub. L. No. 93-406, 88 Stat. 829; Russell Korobkin, *The Battle Over Self-Insured Health Plans, or “One Good Loophole Deserves Another*,*”* 1 Yale J. Health Pol’y L. & Ethics 89, 92–95, 107 (2005) (discussing the structural components of ERISA that provide enhanced protections to self-insured employer health care benefits plans and analyzing the incentives that ERISA gave employers to self-insure).

63
*See* Wendy Silver, Note, *The Inadequacy of State Legislative Responses to ERISA Preemption of Managed Care Liability*, 78 N.Y.U. L. Rev. 845, 851 (2003).

64
*See Employee Retirement Income Security Act (ERISA)*, U.S. Dep’t of Labor, https://www.dol.gov/general/topic/retirement/erisa [https://perma.cc/M42F-A874] (last visited May 10, 2025).

65 McGuire et al., *supra* note 43, at 291, 297.

66 Off. of Pol’y, Soc. Sec. Admin., SSA Pub. No. 13-11700, Annual Statistical Supplement to the Social Security Bulletin, 2006, at 39 (2007).

67 Medicaid & CHIP Payment Access Comm’n, Report to the Congress: The Evolution of Managed Care in Medicaid 117 (2011).

68 Cathy Shoen & Sara R. Collins. *The Big Five Health Insurers’ Membership and Revenue Trends: Implications for Public Policy*, 36 Health Affs. 2185, 2189 (2017).

69
*See* Patient Protection and Affordable Care Act, Pub. L. No. 111-148, 124 Stat. 119 (2010).

70
*See* David K. Jones & Scott L. Greer, *State Politics and the Creation of Health Insurance Exchanges*, 103 Am. J. Pub. Health e8, e8 (2013).

71
*See* Sarah M. Lyon et al., *Medicaid Expansion Under the Affordable Care Act: Implications for Insurance-Related Disparities in Pulmonary, Critical Care, and Sleep*, 11 Annals Am. Thoracic Soc’y 661, 661–62 (2014).

72 Medicaid & CHIP Payment Access Comm’n, March 2015 Report to Congress on Medicaid and CHIP 76–78 (2015).

73
*See* Am. Hosp. Ass’n, Fact Sheet: Majority of Hospital Payments Dependent on Medicare or Medicaid 1–2 (2024); *see also* Anthony W. Orlando & Robert I. Field, *Measuring the COVID-19 Financial Threat to Hospital Markets*, 58 Inquiry art. no. 469580211059985, at 5–6 (2021).

74 Getting the ACA passed in the first place was an enormous challenge and accomplishment for the Obama Administration and was, as Vice President Biden famously said (in a whispered comment that the whole world heard), “a BFD.” *See* Hadas Gold, *Biden Jokes About ‘BFD*,*’* Politico (May 26, 2014), https://www.politico.com/story/2014/05/joe-biden-speech-hot-microphone-107093. But that was just a start, as the law had to withstand the scrutiny of SCOTUS and Donald Trump’s attempts to “repeal and replace Obamacare.” *See generally* Gary Price & Tim Norbeck, *A Look Back at How the President Was Able to Sign Obamacare into Law Four Years Ago*, Forbes (Mar. 26, 2014, 1:27 PM), https://www.forbes.com/sites/physiciansfoundation/2014/03/26/a-look-back-at-how-the-president-was-able-to-sign-obamacare-into-law-four-years-ago/ [https://perma.cc/G5VL-5HSX]; Paul Starr, Remedy and Reaction: The Peculiar American Struggle Over Health Care Reform 201–35 (2011).

75 John Holahan, Urb. Inst., Repeal of the Affordable Care Act: Potential Effects on Coverage, Government Spending, and Provider Revenue 2–3 (2024), https://www.urban.org/sites/default/files/2024-06/Repeal of the Affordable Care Act.pdf [https://perma.cc/UF8H-ATN2].

76
*See, e.g.*, Anna Wilde Mathews, *Elevance Shares Dive After Insurer Faces ‘Unprecedented’ Medicaid Challenge*, Wall St. J. (Oct. 17, 2024, 11:35 AM ET), https://www.wsj.com/health/healthcare/elevancehealth-elv-q3-earnings-report-2024-7dee94ae.

77
*See* Orlando & Field, *supra* note 73, at 6–8.

78 Anna Wilde Mathews et al., *Big Hospitals Provide Skimpy Charity Care-Despite Billions in Tax Breaks*, Wall St. J. (July 25, 2022, 10:26 AM ET), https://www.wsj.com/articles/nonprofit-hospitals-vs-for-profit-charity-care-spending-11657936777.

79 Karyn Schwartz et al., *What We Know About Provider Consolidation*, KFF (Sep. 2, 2020), https://www.kff.org/health-costs/issue-brief/what-we-know-about-provider-consolidation/ [https://perma.cc/5ZUC-9HST].

80
*Id.*

81 Nat’l Council on Comp. Ins. Insights, *The Impact of Hospital Consolidation on Medical Costs*, Nat’l Council on Comp. Ins. (July 11, 2018), https://www.ncci.com/Articles/Pages/II_Insights_QEB_Impact-of-Hospital-Consolidation-on-Medical-Costs.aspx [https://perma.cc/249U-FXLW].

82 Christopher M. Whaley & Xiaoxi Zhao, *The Effects of Physician Vertical Integration on Referral Patterns, Patient Welfare, and Market Dynamics*, 238 J. Pub. Econ. art. no. 105175, at 1–2 (2024).

83 Amy Phillips, *The Consequences of U.S. Hospital Consolidation on Local Economies, Healthcare Providers, and Patients*, Wash. Ctr. for Equitable Growth (Nov. 15, 2023), https://equitablegrowth.org/research-paper/the-consequences-of-u-s-hospital-consolidation-on-local-economies-healthcare-providers-and-patients/ [https://perma.cc/3MEM-SH9A].

84 Eyal Press, *The Moral Crisis of America’s Doctors*, N.Y. Times (July 14, 2023), https://www.nytimes.com/2023/06/15/magazine/doctors-moral-crises.html [https://perma.cc/B3JS-XHTF].

85 Rebecca Anhang Price et al., *Association of Hospice Profit Status with Family Caregivers’ Reported Care Experiences*, 183 JAMA Internal Med. 311, 311, 315–16 (2023).

86
*See* Manuel Adelino et al., *Hospital Financial Health and Clinical Choices: Evidence from the Financial Crisis*, 68 Mgmt. Sci. 2098, 2098–116 (2022).

87
*See* Robert I. Field et al., *Private Equity in Health Care: Barbarians at the Gate?*, 15 Drexel L. Rev. 821, 821–28 (2023).

88
*Id.* at 824, 841.

89
*Id.* at 821–48.

90
*See id.* at 845–46; Sneha Kannan et al., *Changes in Hospital Adverse Events and Patient Outcomes Associated with Private Equity Acquisition*, 330 JAMA 2365, 2366–69 (2023).

91 Elizabeth Schrier et al., *Hospital Assets Before and After Private Equity Acquisition*, 332 JAMA 669, 669–70 (2024).

92
*See, e.g.*, Richard J. Hamilton, *The Hahnemann University Hospital Closure and What Matters: A Department Chair’s Perspective*, 95 Acad. Med. 494, 494–96 (2020).

93 Brendan Ballou, *When Private-Equity Firms Bankrupt Their Own Companies*, Atlantic (May 1, 2023), https://www.theatlantic.com/ideas/archive/2023/05/private-equity-firms-bankruptcies-plunder-book/673896/ [https://perma.cc/4QK4-KQH3].

94 Ashish K. Jha, Opinion, *Private Equity Firms are Gnawing Away at U.S. Health Care*, Wash. Post (Jan. 10, 2024), https://www.washingtonpost.com/opinions/2024/01/10/private-equity-health-care-costs-acquisitions/.

95 Mark Duggan et al., *The Impact of Privatization: Evidence from the Hospital Sector* 3–4, 22–23, 29–31, 38–39 (Nat’l Bureau of Econ. Rsch., Working Paper No. 30824, 2024), https://www.nber.org/system/files/working_papers/w30824/w30824.pdf [https://perma.cc/4YWS-24F3].

96 Larry Pollack, Reason Found., Is Private Equity a Public Financial Hazard? Answering Questions on the Impact of Private Equity Investments on Public Pensions 41 (2024), https://reason.org/wp-content/uploads/is-private-equity-a-public-financial-hazard.pdf [https://perma.cc/7QT3-4UA2].

97
*See, e.g.*, Peter Whoriskey, *Financiers Bought Up Anesthesia Practices, Then Raised Prices*, Wash. Post (June 29, 2023), https://www.washingtonpost.com/business/2023/06/29/private-equity-medical-practices-raise-prices/.

98
*See* Chris Wheat et al., JPMorganChase Inst., The Consistency of Health Insurance Coverage in Small Businesses: Industry Challenges and Insights 7–20 (2025), https://www.jpmorganchase.com/content/dam/jpmc/jpmorgan-chase-and-co/institute/pdf/small-business-health-insurance-report.pdf [https://perma.cc/FU6Y-MJ4J].

99
*See* Pollack, *supra* note 96, at 1, 36; Phillip Levine, *Should College Endowments Be Taxed?*, Brookings Inst. (Sept. 3, 2024), https://www.brookings.edu/articles/should-college-endowments-be-taxed/ [https://perma.cc/GD63-NBTU].

100
*See* Maya Brownstein, *Private Equity’s Appetite for Hospitals May Put Patients at Risk*, Harvard T.H. Chan Sch. of Pub. Health (Dec. 16, 2024), https://hsph.harvard.edu/news/private-equitys-appetite-for-hospitals-may-put-patients-at-risk/ [https://perma.cc/G729-NV4U]; *What Is Carried Interest, and How Is It Taxed?*, Tax Pol’y Ctr. (Jan. 2024), https://taxpolicycenter.org/briefing-book/what-carried-interest-and-should-it-be-taxed-capital-gain [https://perma.cc/P7Z9-N9DW].

101
*See, e.g.*, Whoriskey, *supra* note 97.

102
*See* Coal. for Life Scis., Beyond 2020: A Vision and Pathway for NIH 1–8 (2020), https://www.coalitionforlifesciences.org/beyond-2020-a-vision-and-pathway-for-nih/ [https://perma.cc/8R5S-RNCF].

103 Fernando Carrasco-Ramiro et al., *Human Genomics Projects and Precision Medicine*, 24 Gene Therapy 551, 551, 553 (2017).

104
*See, e.g.*, Nat’l Acad. of Pub. Admin., US Patent and Trademark Office: Transforming to Meet the Challenges of the 21st Century 1–2, 12–13 (2005).

105
*See generally Milestones in U.S. Food and Drug Law*, U.S. Food & Drug Admin. (Jan. 30, 2023), https://www.fda.gov/about-fda/fda-history/milestones-us-food-and-drug-law.

106
*See* Field, *supra* note 6, at 67, 80.

107 Anthony W. Orlando, Letter to the One Percent 57 (2013).

108
*See* Rajiv Leventhal, *Nearly Two-Thirds of US Consumers are Mobile Health App Users*, EMARKETER (Feb. 21, 2023), https://www.emarketer.com/content/nearly-two-thirds-of-us-consumers-mobile-health-app-users [https://perma.cc/B9B3-UCKL].

109
*See At-Home OTC COVID-19 Diagnostic Tests*, U.S. Food & Drug Admin. (Apr. 21, 2025), https://www.fda.gov/medical-devices/coronavirus-covid-19-and-medical-devices/home-otc-covid-19-diagnostic-tests; Debra Fulghum Bruce, *Cholesterol Home Test Kits*, WebMD (Sept. 4, 2023), https://www.webmd.com/cholesterol-management/cholesterol-home-test-kits [https://perma.cc/3YHS-GAAG]; Rachael Ajmera, *Are At-Home Food Sensitivity Tests Reliable? What to Try Instead*, Healthline (Feb. 17, 2025), https://www.healthline.com/health/food-sensitivity-test [https://perma.cc/AT7D-ZB42]; *At-Home Sleep Study*, Sleep Doctor, https://shop.sleepdoctor.com/products/home-sleep-apnea-test [https://perma.cc/R6PN-FTYT] (last visited May 11, 2025).

110
*See* Kyeonghye Guk et al., *Evolution of Wearable Devices with Real-Time Disease Monitoring for Personalized Healthcare*, 9 Nanomaterials 813, 813–14 (2019).

111 Robert I. Field, *The Data We Leave Behind: Limits of Legal Protections for Neurotechnology and Genomic Data*, 15 Drexel L. Rev. 101, 116 (2023).

112
*See* Anthony W. Orlando & Arnold J. Rosoff, *Fast-Forward to the Frightening Future: How the 21st Century Cures Act Accelerates Technological Innovation… at Unknown Risk to Us All*, 44 Am. J.L. & Med. 253, 258 (2018).

113
*See generally* Julia Shaver, *The State of Telehealth Before and After the COVID-19 Pandemic*, 49 Primary Care 517 (2022).

114
*See* Carter H. Nakamoto et al., *The Impact of Telemedicine on Medicare Utilization, Spending, and Quality, 2019–22*, 43 Health Affs. 691, 695 (2024).

115
*See* Mary Reed et al., *Telemedicine Versus In-Person Primary Care: Treatment and Follow-Up Visits*, 176 Annals Internal Med. 1349, 1352–55 (2023).

116
*See generally* Kimberly Cornaggia et al., *Financial Effects of Remote Product Delivery: Evidence from Hospitals*, 37 Rev. Fin. Stud. 2817 (2023).

117 The FDA’s approach is reflected in its Digital Health Innovation Action Plan, issued in 2017, and its Guidance on Mobile Medical Applications, last updated in 2022. *See generally* U.S. Food & Drug Admin., Digital Health Innovation Action Plan (2017), https://www.fda.gov/media/106331/download; U.S. Food & Drug Admin., Policy for Device Software Functions and Mobile Medical Applications (2022), https://www.fda.gov/media/80958/download. Congress’s attitude is reflected in the 21st Century Cures Act. *See generally* 21st Century Cures Act, Pub L. No. 114-255, 130 Stat. 1033 (2016); *21st Century Cures Act: Key Provisions Related to Medical Devices* 6–8, Covington (July 30, 2015), https://www.cov.com/-/media/files/corporate/publications/2015/07/21st_century_cures_act.pdf [https://perma.cc/2DN6-ADXT].

118
*See* Anthony W. Orlando & Arnold J. Rosoff, *The New Privacy Crisis: What’s Health Got to Do with It?*, 132 Am. J. Med. 127, 127–28 (2019).

119 Shuroug A. Alowais et al., *Revolutionizing Healthcare: The Role of Artificial Intelligence in Clinical Practice*, 23 BMC Med. Educ. art. no. 689, at 3–7 (2023).

120
*See* Swaroop Aradhya et al., *Applications of Artificial Intelligence in Clinical Laboratory Genomics*, 193 Am. J. Med. Genetics Part C art. no. e32057, at 2–10 (2023).

121
*See* Seema Yelne et al., *Harnessing the Power of AI: A Comprehensive Review of Its Impact and Challenges in Nursing Science and Healthcare*, 15 Cureus art. no. e49252, at 2 (2023).

122
*See* Nazish Khalid et al., *Privacy-Preserving Artificial Intelligence in Healthcare: Techniques and Applications*, 158 Computs. Biology & Med. art. no. 106848, at 2, 6–9 (2023).

123
*See* Richard J. Chen et al., *Algorithmic Fairness in Artificial Intelligence for Medicine and Healthcare*, 7 Nature Biomed. Eng’g 719, 720 (2023); *see also* Robert I. Field, Anthony W. Orlando & Arnold J. Rosoff, *Demographic Diversity of Genetic Databases Used in Alzheimer’s Disease Research*, 142 Hum. Genetics 1215 (2023).

124
*See, e.g.*, *AI in 2024: US Announces $100 million toward R&D as OpenAI Breaks VC Funding Records*, Boast (Oct. 3, 2024), https://boast.ai/blog/ai-in-2024-us-announces-100-million-toward-rd-as-openai-breaks-vc-funding-records/ [https://perma.cc/3KKT-PTZM].

125
*See* Jacob Larson et al., *The Evolution of Artificial Intelligence (AI) Spending by the U.S. Government*, Brookings Inst. (Mar. 26, 2024), https://www.brookings.edu/articles/the-evolution-of-artificial-intelligence-ai-spending-by-the-u-s-government/ [https://perma.cc/T8R3-QM97].

126
*See, e.g.*, Robert I. Field, Anthony W. Orlando & Arnold J. Rosoff, *Am I My Cousin’s Keeper? A Proposal to Protect Relatives of Genetic Database Subjects*, 18 Ind. Health L. Rev. 1, 6–7, 17–22 (2021).

